# Identification and verification of ultrafine particle affinity zones in urban neighbourhoods: sample design and data pre-processing

**DOI:** 10.1186/1476-069X-8-S1-S5

**Published:** 2009-12-21

**Authors:** Paul Harris, Sarah Lindley, Martin Gallagher, Raymond Agius

**Affiliations:** 1 National Centre for Geocomputation, National University of Ireland, Maynooth, Ireland, UK; 2 School of Environment & Development (Geography), University of Manchester, UK; 3 Centre for Atmospheric Science, School of Earth, Atmospheric & Environmental Science, University of Manchester, UK; 4 Occupational and Environmental Health Research Group, School of Translational Medicine, University of Manchester, UK

## Abstract

A methodology is presented and validated through which long-term fixed site air quality measurements are used to characterise and remove temporal signals in sample-based measurements which have good spatial coverage but poor temporal resolution. The work has been carried out specifically to provide a spatial dataset of atmospheric ultrafine particle (UFP < 100 nm) data for ongoing epidemiologic cohort analysis but the method is readily transferable to wider epidemiologic investigations and research into the health effects of other pollutant species.

## Introduction

Urban sources of atmospheric ultrafine particles (UFPs < 100 nm) are largely traffic related and resultant number concentrations exhibit high temporal and spatial variability [1-3]. Although some progress has been made in understanding UFP spatial and temporal dependencies, and models are beginning to emerge, knowledge of UFP dispersion behaviour does not yet support the development of fully robust dispersion algorithms [4]. Since epidemiological studies require but often lack, valid and reliable exposure information taking into account both temporal and spatial variation, research into the wider health outcomes of UFPs is currently restricted [2]. Such work is important due to the increasing evidence of the higher mass-for-mass toxicity of UFPs compared to larger particles [5,2].

Existing experimental work has tended to rely on analysis of data at a small number of locations often creating rich temporal datasets but more limited spatial datasets [3]. However, the emphasis in this study is on the creation of a large spatial dataset of spot samples which can then be linked to exposure relevant spatial units. Since concentrations reflect both spatial and temporal processes, a first task was to design a means of removing temporal signals from the data. This article therefore details sample design and a novel data pre-processing approach, which provides input for ultimate research aims of: (a) UFP local variance estimation for affinity zone identification (exposure-relevant zones within which UFP concentrations are likely to be similar) and (b) UFP prediction for exposure characterisation.

## Methodology

### Sample design and data collection

**Figure 1:**
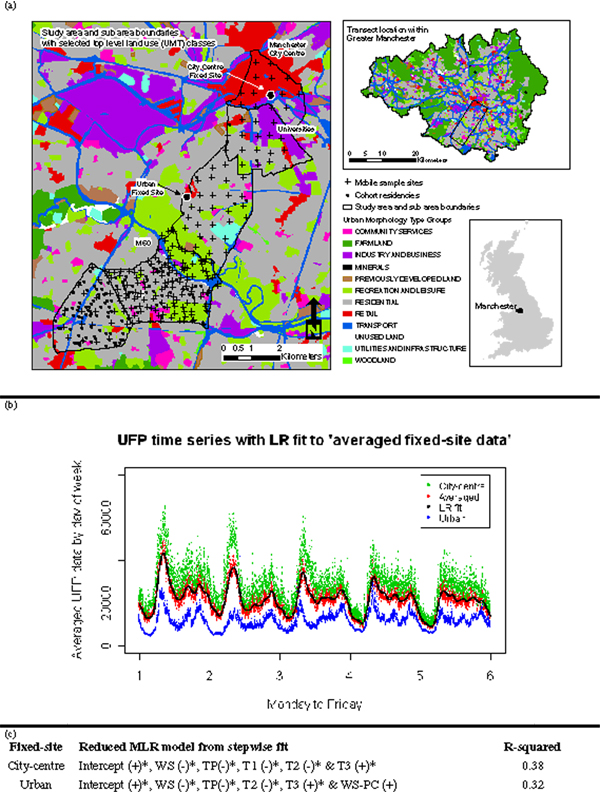
**a) study area; b) LR fit to an average of both fixed-site datasets, themselves averaged by day; c) Summary of MLR fits to hourly fixed-site datasets**. MLR fits are given with the direction of the relationship and covariates marked with * indicate that their coefficients are significantly different from zero at the 99.9% level.

Sampling occurred from mid January to mid May 2008 and had a fixed and a mobile component. The fixed component comprised a city-centre and an urban background site (Figure 1a) and was intended to: (a) provide a broad understanding of temporal variation in UFP in two different settings within the study area and (b) enable pre-processing of the mobile data. UFP one minute mean number concentration counts were taken continuously throughout the five-month sampling period. After quality control, usable data covered a 14 week period. The mobile component comprised 232 3-minute mean spot measurements made over a 32 km^2 ^transect from Manchester city centre to the suburbs. The transect-based study area was a compromise between the need to maximise coverage of: land-use types; distances from the city centre; and cohort residences within an ongoing asthma and allergy birth cohort [6]. Mobile sites were chosen at two different spatial resolutions. The first was designed to help capture large (city) scale patterns and involved making spot measurements using a pseudo-regular grid. To aid the logistics of spatial sampling five sub-areas were used. The second was designed to allow the consideration of medium scale patterns and used a mixture of random and stratified random sampling (by land-use zone) in one sub-area (Figure 1a).

All mobile-site data were collected on weekdays 10 am-3 pm under mainly dry and wind-free meteorological conditions. This targeted sampling campaign was chosen so as to minimise unwanted temporal and meteorological variability (particularly wind and precipitation) so that the data could be considered a snapshot of a UFP *spatial *process. An average of 25 sites was visited on any given sampling day and duplicate measurements were found at most sites for later cross validation. All sites had at least one UFP measurement and the final dataset consisted of 402 measurements in total. For both fixed- and mobile-site UFP data, corresponding meteorological data were also found. For the former, Met Office data were used, and for the latter local meteorological measurements were found using a hand-held (kestrel ^®^) device. UFP measurements were found using TSI ^® ^WCPCs (model 3781) at the fixed-sites and hand-held TSI ^® ^P-TRAKs (model 8525) at the mobile-sites both of which have high sampling rates.

### Pre-processing the UFP data

Even with a targeted sampling campaign, some unwanted temporally and meteorologically induced variability in the mobile-site UFP data is expected. Consequently, this data is pre-processed to produce *spatial *data more suitable for research aims. Air pollution studies that have used some sort of data pre-processing prior to a predictive regression fit include those of [7-10], but none provided a methodology transferable to this research. As such, a novel pre-processing methodology is presented and involves an investigation of patterns in the fixed-site UFP data at two temporal scales.

Small-scale diurnal trends in the fixed-site UFP data were analysed in order to provide the basis for pre-processing the mobile-site data to an equivalent time of day between the 10 am-3 pm sampling frame. A nonlinear, local regression (LR) fit [11] was found for individual averaged weekdays. It was assumed that this LR fit represents the same underlying diurnal pattern in UFP variability at each mobile-site and adjustments could therefore be made assuming a proportional relationship.

Wintertime conditions seem to provide highest UFP concentrations and relationships have also been found with temperature and wind speed [12,13]. Therefore it was also necessary to carry out large-scale, temporal and meteorological mobile-site data pre-processing. This accorded to separate multiple linear regression (MLR) fits to each fixed-site UFP dataset, specified with three temporal indicator covariates (day of week (T1), sampling week (T2) and day/night (T3)); two meteorological continuous covariates (wind speed (WS) and temperature (TP)); and two meteorological indicator covariates (precipitation (PC) and a WS-PC interaction term). This provided two forms of large-scale data pre-processing results: a *detrended *and an *adjusted *UFP spatial dataset.

### Validation of pre-processing strategy

Duplicate mobile-site samples were earmarked for cross validation and then screened resulting in 169 duplicate measurements for 169 sites. This data was then divided into two equally-sized cross-validation datasets, A and B.

## Results

### Pre-processing step 1: small-scale adjustments

Average diurnal patterns in the fixed-site UFP data indicates higher concentration counts and higher variability at the city-centre site compared to the urban site (Figure 1b). An LR fit to an average of the two time-series datasets was used for the small-scale UFP adjustments (Figure 1b with a 4-hour smoothing bandwidth). Mobile-site UFP data can now be adjusted to reflect sampling at a user-specified time of 12 pm using: . Here UFP_ADJ_SS and UFP_ACT are the adjusted and actual values, respectively; and UFP_PRED_1 and UFP_PRED_2 are LR predictions found at the actual sample time (e.g. 11.09 am) and at 12 pm, respectively. UFP_PRED_1 and UFP_PRED_2 must correspond to the weekday that UFP_ACT was sampled on.

### Pre-processing step 2 (option A): large-scale detrending

For Step 2, a MLR model is fitted to each of the fixed-site datasets, where UFP and meteorological measurements are taken in a 'raw' hourly-averaged form. A stepwise fitting procedure is followed and the reduced MLR fits retain five of the original seven UFP covariates (Figure 1c). Each covariate only relates to variation that needs to be filtered from the small-scale adjusted data (Step 1). In this respect, the resultant regressions are not expected to fit well, as the underlying sources of UFP are not being modelled. Next the mobile-site UFP data is detrended using one of the fixed-site regressions according to whether a mobile site is classed as urban or city centre (based on proximity). For clarity, a mobile-site detrending involves; (i) specifying one of the fixed-site regressions with covariate data particular to the mobile-site, (ii) calculating the UFP prediction and (iii) subtracting this prediction from the actual mobile-site UFP value. The key assumption is that the coefficients of the fixed-site regressions are *transferable *to the mobile-sites.

### Pre-processing step 2 (option B): large-scale adjustments

Detrended data of step 2 (option A) provides pre-processed data loosely centred on a mean of zero, which is suitable for modelling UFP variability but unsuitable for UFP prediction. Consequently, the (small-scale adjusted) mobile-site data of step 1 is adjusted a second time to provide a *fully-adjusted *pre-processed dataset. These large-scale adjustments are directly based on the fixed-site detrending regressions and are similar in concept to the small-scale adjustments of step 1, i.e. proportionality is assumed. In this case, user-specified UFP covariate values are chosen so that adjusted datasets can be found for any particular time (for T1, T2 and T3) and meteorological condition (for WS, TP and WS-PC). This could be such that the *lowest*, *average*, or *highest *expected UFP concentrations are found. For this article, an adjusted dataset is found where *average *UFP concentrations are expected.

### Cross-validation

**Figure 2:**
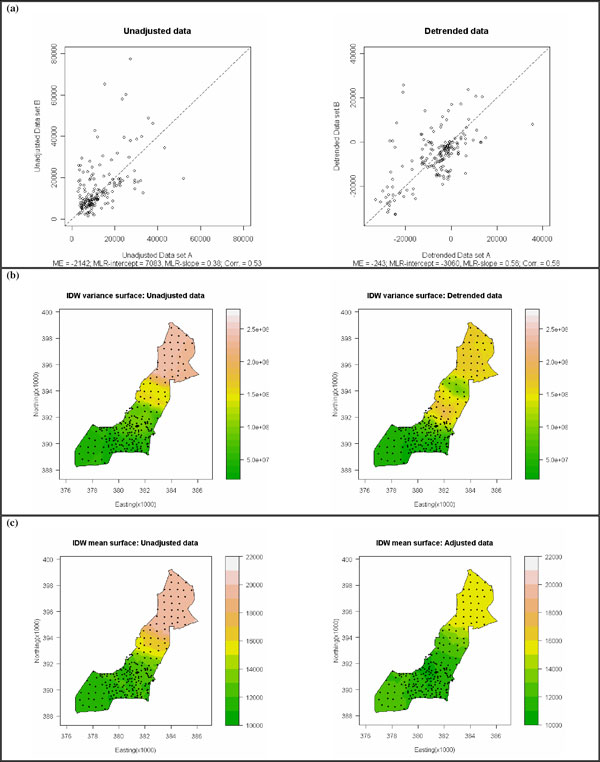
**a) cross-validation scatterplots and diagnostics; b) local UFP variance surfaces; c) local UFP mean surfaces**.

Original unadjusted data and pre-processed data are both affected by spatial drivers of UFP concentration patterns. However, since unwanted temporal and meteorological influences have been removed in the pre-processed data, pre-processed cross-validation dataset A should relate more strongly to pre-processed cross-validation dataset B, than is the case for the unadjusted cross validation datasets. Therefore our pre-processing strategy can be validated by using scatterplots and associated diagnostics (Figure 2a). A strong relationship between the two cross-validation datasets should result in a mean error () of zero (reflecting an even scatter around the 45° line), an MLR-intercept of zero, an MLR-slope of one and a correlation coefficient of 1. All diagnostics indicate that the detrended pre-processed data performs better than the unadjusted data and as such should be preferred for subsequent research modelling aims. Similarly positive cross-validation results were found for the adjusted pre-processed data.

## Exploratory analysis for the modelling stages of the research

Exploratory investigations are presented to clarify the value of the pre-processing methodology with respect to the dual aims of this research.

### UFP local variance estimation

Geographically weighted local variance surfaces [14] are presented for unadjusted and detrended UFP data in Figure 2b. The local variances are found using an inverse distance weighting (IDW) function (bandwidth = 25%). As expected, both surfaces indicate areas of high and low UFP variability in the city-centre and suburban areas, respectively. Alternative measures of local variability and different optimisation algorithms are being developed, building on existing work in this area [15].

### UFP prediction

Relationships between UFP and a number of urban covariates are also being explored and can be used to provide input into a land-use regression approach [16]. Covariates include road and traffic patterns, population density, building heights, distance to city centre, exposure-relevant land-use zones and associated surface cover properties. Depending on the nature of any spatial dependence found in the UFP data, these covariates may also provide input to a geostatistical approach [17]. Figure 2c shows exploratory IDW mean surfaces for spatial trends (bandwidth = 35%). The unadjusted and adjusted data exhibit a clear spatial trend from the city centre to the suburbs, the nature of which is entirely reasonable and suggestive of city-scale patterns, which may underlie the metre scale variability reported elsewhere [1]. The adjusted data has a much reduced UFP variance over the unadjusted data, partially reflecting its *on average *specification.

## Conclusion

This article presented and validated a methodology to pre-process highly variable UFP number concentration data collected through spot sampling into data that can be assumed to represent data collected at the same time and under the same meteorological conditions. This cost-effective approach to sampling provides an information-rich spatial dataset that is rarely found in UFP studies or wider air pollution research.

The study whose preliminary methods have been described here will form the basis of more robust epidemiologic research for cohort studies a large proportion of whose population reside in the study area described in [6]. Moreover, the methodology from this study is transferable to other epidemiologic investigations and should permit a better classification of exposure as well as a more efficient sampling strategy.

## List of abbreviations used

UFP: Ultra Fine Particles; MLR: multiple linear regression; LR: local regression; IDW: inverse distance weighting; UMT: Urban Morphology Unit (land-use class).

## Note

The peer review form to this article can be found in Additional file 1.

## Competing interests

The authors declare that they have no competing interests.

## Authors' contributions

The original study was conceived by MG, RA and SL. SL and PH collaborated on detailed study design which included specific inputs from PH. PH carried out the major part of data collection, all of the statistical analysis and drafted the manuscript. SL contributed to manuscript writing with some input from RA and MG. All authors read and approved the final manuscript.

## Supplementary Material

Additional file 1 Peer review Click here for file
