# Supplementary material for: Identification and verification of ultrafine particle affinity zones in urban neighbourhoods: sample design and data pre-processing
Source: Environ Health. 2009 Dec 21;8(Suppl 1):S5. doi: 10.1186/1476-069X-8-S1-S5 (PMC2796501; doi:10.1186/1476-069X-8-S1-S5)
Supplement: Additional file 1 — Peer review [file 1476-069X-8-S1-S5-S1.doc]

| Article ref no. | A1471-2105X SZ S5 |
| --- | --- |
| Title | **Identification and Verification of Ultrafine Particle Affinity Zones in Urban Neighbourhoods: Sample Design and Data Post-processing** |
| Author(s) | Paul Harris, Sarah Lindley, Martin Gallagher, Raymond Agius |

**Ref#1**

**General comments:**

The manuscript introduces a new methodology for the processing of atmospheric ultrafine particle datasets. This is an interesting and well written paper.

**Major compulsory revisions:**

My only major concern is that there is no information about the methodology used to sample UFPs. How were they actually collected? Also, some information is provided about the size of the area and the timing of data collection for the mobile site but not for the fixed site. Given the nature of the manuscript I feel this information is essential to be added.

**Minor essential revisions:**

None

**Ref#2**

**General comments:**

This is an important and potentially very useful paper for the further development of this field and is recommended for publication unchanged.

**Authors’ response**

The authors welcome the positive comments on the importance of the paper. The paper reports findings from one component of a proof of concept study funded under the Joint Environment and Human Health Programme (NE/E009565/1) with other outputs to follow. The methodology used to collect number concentration data was originally given in brief in the sample design section. The title of the section has been revised and more detail given on the data collection methodology and its rationale, particularly for the fixed stations. The final modification has been to clarify that the developed methodology is strictly more correctly termed as being associated with data pre-processing rather than data post-processing. This is because in the context of the project within which it was developed, the methodology was required in order to produce a robust spatial dataset suitable for subsequent spatial analysis.
